# Lipoprotein particle distribution and skeletal muscle lipoprotein lipase activity after acute exercise

**DOI:** 10.1186/1476-511X-11-64

**Published:** 2012-07-10

**Authors:** Michael Harrison, Niall M Moyna, Theodore W Zderic, Donal J O’Gorman, Noel McCaffrey, Brian P Carson, Marc T Hamilton

**Affiliations:** 1 Department of Health, Sport and Exercise Science, Waterford Institute of Technology, Waterford, Ireland; 2 Centre for Preventive Medicine and School of Health and Human Performance, Dublin City University, Dublin, Ireland; 3 Inactivity Physiology Department, Pennington Biomedical Research Center, Perkins Road, Baton Rouge, LA, 70808, USA; 4 Department of Physical Education and Sports Science, University of Limerick, Limerick, Ireland

**Keywords:** Exercise, Lipoprotein lipase, Very low density lipoprotein, Triglyceride, Lipoprotein size, Energy deficit

## Abstract

**Background:**

Many of the metabolic effects of exercise are due to the most recent exercise session. With recent advances in nuclear magnetic resonance spectroscopy (NMRS), it is possible to gain insight about which lipoprotein particles are responsible for mediating exercise effects.

**Methods:**

Using a randomized cross-over design, very low density lipoprotein (VLDL) responses were evaluated in eight men on the morning after i) an inactive control trial (CON), ii) exercising vigorously on the prior evening for 100 min followed by fasting overnight to maintain an energy and carbohydrate deficit (EX-DEF), and iii) after the same exercise session followed by carbohydrate intake to restore muscle glycogen and carbohydrate balance (EX-BAL).

**Results:**

The intermediate, low and high density lipoprotein particle concentrations did not differ between trials. Fasting triglyceride (TG) determined biochemically, and mean VLDL size were lower in EX-DEF but not in EX-BAL compared to CON, primarily due to a reduction in VLDL-TG in the 70–120 nm (large) particle range. In contrast, VLDL-TG was lower in both EX-DEF and EX-BAL compared to CON in the 43–55 nm (medium) particle range. VLDL-TG in smaller particles (29–43 nm) was unaffected by exercise. Because the majority of VLDL particles were in this smallest size range and resistant to change, total VLDL particle concentration was not different between any of these conditions. Skeletal muscle lipoprotein lipase (LPL) activity was also not different across these 3 trials. However, in CON only, the inter-individual differences in LPL activity were inversely correlated with fasting TG, VLDL-TG, total, large and small VLDL particle concentration and VLDL size, indicating a regulatory role for LPL in the non-exercised state.

**Conclusions:**

These findings reveal a high level of differential regulation between different sized triglyceride-rich lipoproteins following exercise and feeding, in the absence of changes in LPL activity.

## Introduction

Single sessions of exercise transiently reduce serum triglycerides (TG). This exercise effect is not always apparent immediately post-exercise, it can occur after a delay of hours and is generally maximal on the day following intense and prolonged exercise [1,2]. Reductions in serum triglycerides of 18 – 22% are typically observed on the morning after a prolonged exercise bout [3]. This effect is blunted when the energy [4] and carbohydrate [5] deficit induced by exercise is replaced during the hours post-exercise. Thus, studies of acute exercise on lipids and lipoproteins should ideally control for energy and carbohydrate intake in order to distinguish the effects of exercise and energy expenditure *per se* from the effects of an exercise-induced energy or carbohydrate deficit.

Classic lipid panels measure only the total amount of TG in plasma or cholesterol in low (LDL) or high (HDL) density lipoproteins. However, more advanced approaches are now capable of quantifying the concentration of LDL, HDL, very low density lipoprotein (VLDL) and intermediate density lipoprotein (IDL) particles of different size populations. Importantly, the nuclear magnetic resonance spectroscopy (NMRS) determined lipoprotein size of particles and the concentration of specific lipoprotein particles have been associated with risk of diabetes [6] and cardiovascular disease [7,8] independent of typical biochemical assessments of TG and cholesterol. For example, large VLDL particles and small LDL particles were associated with greater prospective risk of type 2 diabetes after adjusting for standard lipids and other non-lipid risk factors [6]. Defects in metabolism of large VLDL particles tend to drive the metabolic reactions producing small, dense pro-atherogenic LDL particles of abnormal size in tandem with lowering of anti-atherogenic HDL [9]. To date, few studies have examined the influence of acute exercise on NMRS-determined VLDL lipoprotein particles.

Although understanding of the mechanisms underpinning the hypotriglyceridemic effects of exercise has advanced considerably in recent years, much remains to be learned [10]. Post-exercise hypotriglyceridemia has frequently been linked to increased skeletal muscle lipoprotein lipase (LPL) activity. However the evidence supporting this assertion is not compelling, particularly on the morning after exercise when serum TG are considerably reduced. Indeed, only two studies have examined LPL activity at this timepoint, with one showing an increase [11] and one showing no change [12]. The need to consider energy balance in exercise studies examining TG metabolism has been recently identified [10]. This may not have been fully appreciated in previous LPL studies, potentially accounting for discrepant results. More recently, research attention has focused on VLDL composition. Data from kinetic studies [13,14] suggest that the liver secretes fewer but more triglyceride rich VLDL particles on the morning after prolonged exercise, increasing particle affinity with LPL. These studies underline the potential of changes in VLDL particles to influence TG clearance by mechanisms other than LPL.

The purpose of this study was therefore to examine the influence of prolonged acute exercise on serum lipids, NMRS-determined lipoprotein subfractions and LPL activity in skeletal muscle. These assessments were undertaken ~12 h post-exercise when serum TG are known to be considerably reduced. The results of this study will for the first time quantify the influence of acute exercise on the size and concentration of 24 different VLDL subfractions. They will also clarify the effects of exercise on LPL activity in skeletal muscle and the role of this enzyme in mediating any observed changes in lipoprotein particles. The study involved two exercise trials, one with and one without carbohydrate replacement, in order to distinguish the effects of exercise *per se* from an exercise-induced energy deficit.

## Methodology

### Subjects

Eight moderately active men (mean ± SD; age 26.9 ± 4.1 y, VO_2_peak 46.8 ± 4.9 mL^.^kg^-1.^min^-1^, body mass, 83.5 ± 13.7 kg BMI, 26.0 ± 3.6 kg^.^m^-2^ body fat 15.2 ± 5.0%) volunteered for this study. Subjects were non-smokers, normolipidemic, free from cardiovascular disease and diabetes and not taking medication known to influence carbohydrate or lipid metabolism. Ethical approval was granted by the Dublin City University Research Ethics Committee and the Pennington Biomedical Research Center Institutional Review Boards. Written informed consent was also obtained. These methods and other data have been described in greater detail elsewhere [5].

### Experimental design and exercise bouts

On three test mornings, separated by 7 d approximately, subjects underwent a muscle biopsy followed by a blood sample for determination of lipids and lipoproteins. On the evening prior to one test morning, subjects rested quietly at home (CON). On the evening prior to two test mornings, subjects exercised in the laboratory. Following one of these exercise sessions, glucose was consumed to replete muscle glycogen and restore energy and carbohydrate balance (EX-BAL). Following the other exercise session (EX-DEF), only water was permitted, thus maintaining a CHO deficit and low muscle glycogen concentration. These trials were undertaken in random order. The experimental exercise sessions were conducted between 17.00 h and 19.00 h on the evening prior to the muscle biopsy and fasting blood sample. Subjects cycled for 90 min at a load equaling 70% VO_2_peak, followed by ten 1 min full effort sprints interspersed with 1 min of resting recovery. Expired air was collected continuously during the continuous cycling via a breath by breath metabolic system (Vmax 229, Sensormedics, Yorba Linda, CA). Energy expenditure and substrate oxidation was estimated using indirect calorimetry [15]. Energy expenditure during the sprints was estimated from an ACSM metabolic equation [16] based on flywheel resistance and revolutions.

### Dietary control

Diet was strictly standardized with subjects consuming 3 meals provided by the laboratory on the day prior to each test morning. These were consumed in all 3 trials at approximately 08.00, 12.00 and 16.00 h. This pre-test diet fed before each trial consisted of standardized mixed meals to provide 56% carbohydrate, 14% protein, and 30% fat. Subjects were required to abstain from alcohol and not to engage in exercise or heavy physical work outside of laboratory testing for 3 days prior to each trial. Subjects were reminded of these diet and activity requirements during phone contact in advance of each subsequent test day. Compliance was checked verbally.

### Carbohydrate replacement after intense exercise (EX-BAL)

In addition to the 3 standardized meals in each of the 3 trials, in one of the exercise conditions (EX-BAL) carbohydrate was provided with the intended purpose of restoring glycogen concentration. During the evening after exercise, subjects ingested 105% of the CHO oxidized during exercise, (4.4 ± 0.2 g.kg^-1^) and 94% of the total exercise energy expenditure. The intention was to restore both CHO and energy balance, while avoiding muscle glycogen supercompensation. Water was consumed at equivalent time-points in CON and EX-DEF. From indirect calorimetry, we calculated that 349 ± 17 grams of CHO were oxidized and the total energy expenditure during the exercise session was 1,507 ± 75 kcals. The replacement averaged 367 ± 18 grams CHO and 1,420 ± 69 kcals. This was achieved with an 18% CHO drink and 85% glucose confectionary consumed at 0, 2, and 4 hours after exercise, and then fasted for 10 hours.

### Muscle biopsy and blood sampling

Subjects travelled to the laboratory on each test morning by motorized transport. They had been fasting for at least 10 h. A muscle biopsy was obtained from the midway point of the vastus lateralis after local anesthesia with 2% lidocaine. Biopsies were obtained from the same leg for the first and third trial and from the alternate leg for the second trial. Samples were snap frozen in liquid nitrogen and stored at −80°C until analysis. A fasting blood sample was obtained from a prominent forearm vein.

### Lipoprotein lipase assay

Lipoprotein lipase is an enzyme required for catabolyzing plasma triglyceride and generating fatty acids. A stable, radioactive substrate emulsion assay as originally developed [17] has been used extensively for examining skeletal muscle LPL activity responses to physical activity [18-20]. Muscle biopsy samples were homogenized in a buffer containing heparin (5 U.mL^-1^), 0.05 M Tris●HCl (pH 8.1), aprotinin (2 μg.mL^-1^), leupeptin (10 μg.mL^-1^), benzamidine (1 mM), pepstatin (1 μg.mL^-1^), EDTA (5 mM), and BSA (1 mg.mL^-1^) at a concentration of 10 mg of muscle per 350 μL buffer. LPL activity was measured by the rate of hydrolysis of a ^3^H] triolein containing substrate emulsified with lecithin, in the presence of pooled heat-inactivated human serum as the source of apolipoprotein C-II, fatty acid-free albumin and heparin. Assays were performed at 37°C for 100 min. One data point is missing in the EX-DEF and EX-BAL trials due to insufficient muscle. Serial dilutions of representative samples verified that the LPL assay was linear with time and amount of enzyme in the range that assays were performed.

### Glycogen assay

Glycogen concentrations were determined in duplicate by a standard enzymatic technique [21] with fluorometric detection. Briefly, 2 mg of freeze-dried muscle was brought to −15°C from −80°C in a freezer. Samples were incubated in 0.5 mL of 2 N hydrochloric acid for 2 h at 100°C and then reconstituted to original weight with distilled H_2_O, before being neutralized with 1.5 mL of 0.67 N NaOH. One mL of reagent mix containing Tris base (50 mM), HCl (25 mM), MgCl_2_ (1 mM), DTT (0.5 mM), ATP (0.3 mM), NADP (0.05 mM), hexokinase (1 U.mL^-1^), and glucose-6-phosphate dehydrogenase (0.1 U.mL^-1^) was added to samples and glycogen content determined fluorometrically.

### Lipid and lipoprotein subclass analysis

**Figure 1:**
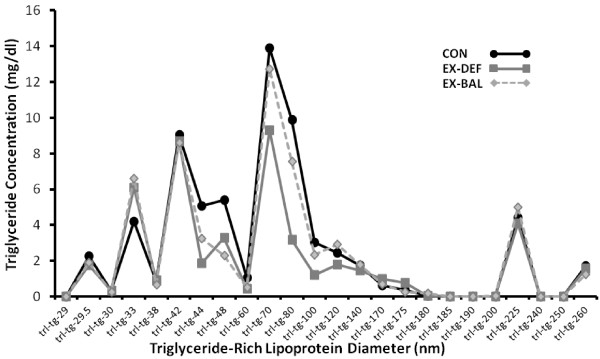
Fasting very low density lipoprotein-triglyceride (VLDL-TG) in 24 different particle size subfractions in CON, EX-DEF and EX-BAL, determined by nuclear magnetic resonance spectroscopy.

Serum was centrifuged at 1600 g for 15 min at 4°C. Total serum TG were determined using a spectrophotometric assay, performed on an automated bench-top clinical chemistry system (ACE®, Alfa Wassermann B.V., Netherlands), using appropriate reagents, calibrators and controls (Randox Laboratories, UK). Lipoprotein subclass analysis was performed from EDTA plasma through the use of nuclear magnetic resonance spectroscopy (NMRS) (LipoScience, Inc. Raleigh, NC). The particle concentration of each lipoprotein class was determined by measuring the amplitude of the lipid methyl groups which are distinct to each size and class [22]. Conversion factors then relate these signal amplitudes to either particle concentration or lipid mass concentration units in conjunction with purified subclass lipid standards. The following 9 lipoprotein subclass categories were investigated in fasting plasma samples for particle concentration: large VLDL (55 to 260 nm), medium VLDL (43 to 55 nm), small VLDL (29 to 43 nm), IDL (23 to 27 nm), large LDL (21.2 to 23 nm), small LDL (18 to 21.2 nm), large HDL (8.8 to 13 nm), medium HDL (8.2 to 8.8 nm), and small HDL (7.3 to 8.2 nm). VLDL and LDL subclass particle concentrations are reported in units of nanomoles per liter and those of HDL subclasses in micromoles per liter. In addition to the three above broad VLDL subclasses, termed small, medium and large according to Liposcience cutoffs, triglyceride mass was described in 24 smaller VLDL subfractions (VLDL-TG). This detailed breakdown was examined to identify the particular ranges of VLDL in which TG is concentrated and the ranges that were most sensitive to exercise (Figure 1).

### Data analysis

Between trial difference were determined using a one-way repeated measures analysis of variance (ANOVA) followed by Fisher Least Significant Difference post-hoc tests. The TG and VLDL lipoprotein particle data were not normally distributed and so were log-transformed prior to analysis. Associations between selected variables were determined using Spearman rank order correlations. Data are reported as mean ± SEM. Statistical significance was set at p < 0.05.

## Results

### Exercise bouts

The workrates (197 ± 9 vs 197 ± 9 W), VO2 (33.4 ± 1.1 vs 33.2 ± 1.0 mL.kg^-1^.min^-1^), respiratory exchange ratios (0.96 ± 0.005 vs 0.96 ± 0.004) and heart rates (151 ± 3 vs 150 ± 4 b.min^-1^) during the 90 min of continuous cycling did not differ in EX-DEF and EX-BAL, respectively. Muscle glycogen was 2.3-fold higher on the morning following the EX-BAL bout compared to the EX-DEF bout (300 ± 23 vs. 128 ± 16 mmol.kg^-1^ DW, p < 0.05). The EX-BAL and CON (300 ± 23 vs. 316 ± 15 mmol.kg^-1^ DW, p = 0.18) values were not significantly different.

### Lipoprotein particles in CON, EX-DEF and EX-BAL

In all trials, VLDL particle concentration was highest in the small (29 – 43 nm) VLDL fraction (Table 1). The majority of the TG mass (VLDL-TG) was distributed in the larger particles of ~ 70–120 nm (Figure 1). Serum TG, determined biochemically, was lower (p < 0.05) in EX-DEF but not in EX-BAL compared to CON (Table 1). Large VLDL particle concentration was lower (p < 0.05) in EX-DEF but not in EX-BAL compared to CON with medium VLDL particle concentration lower (p < 0.05) in EX-DEF and EX-BAL compared to CON (Table 1). Small and total VLDL particle concentrations were not different across trials (Table 1). Mean VLDL particle size was smaller (p < 0.05) in EX-DEF, but not in EX-BAL compared to CON. IDL, LDL and HDL particle concentration and size were not different across trials (Table 1).

**Table 1:** Fasting plasma lipoprotein particle profile in CON, EX-DEF and EX-BAL

	**CON**	**EX-DEF**	**EX-BAL**
□ Total triglyceride (mg/dL)	83.9 ± 12.6	56.5 ± 6.9 *	77.1 ± 5.2**
*VLDL TG mass* (mg/dl)			
□ Total VLDL-TG	66.6 ± 9.2	47.8 ± 5.6	58.9 ± 3.8
□ Large VLDL-TG (55–260 nm)	39.3 ± 8.2	24.8 ± 3.6 *	35.3 ± 2.8**
□ Large VLDL-TG (70–120 nm)	29.3 ± 7.4	15.5 ± 3.3 *	25.6 ± 2.6**
□ Medium VLDL-TG	10.5 ± 1.0	5.2 ± 2.3 *	5.6 ± 1.3 *
□ Small VLDL-TG	16.8 ± 1.4	17.8 ± 1.6	18.1 ± 2.4
*VLDL particle concentration* (nmol/L)			
□ Total VLDL	41.3 ± 3.4	37.9 ± 3.5	40.5 ± 3.5
□ Large VLDL (55–260 nm)	4.1 ± 0.8	2.2 ± 0.5*	3.5 ± 0.4**
□ Medium VLDL	7.5 ± 0.7	3.7 ± 1.6*	4.0 ± 0.9*
□ Small VLDL	29.7 ± 2.4	32.0 ± 3.1	33.0 ± 3.6
*LDL particle concentration* (nmol/L)			
Total LDL	698 ± 64	702 ± 49	682 ± 56
Large LDL	205 ± 53	235 ± 69	175 ± 38
All Small LDL	299 ± 37	297 ± 49	312 ± 15
IDL	194 ± 24	170 ± 19	195 ± 27
*HDL particle concentration* (μmol/L)			
Total HDL	23.9 ± 0.9	24.3 ± 1.4	23.9 ± 1.1
Large HDL	2.2 ± 0.2	2.5 ± 0.4	2.1 ± 0.31
Medium HDL	7.4 ± 0.8	7.7 ± 1.1	6.9 ± 0.97
Small HDL	14.3 ± 0.5	14.0 ± 0.8	14.9 ± 1.1
*Mean Particle Size* (nm)			
□ VLDL	51.8 ± 1.8	46.0 ± 1.9*	50.9 ± 1.5**
LDL	20.8 ± 0.2	20.7 ± 0.2	20.7 ± 0.1
HDL	9.0 ± 0.1	9.1 ± 0.1	9.0 ± 0.2

Values are mean ± SEM; *n* = 8. CON, non-exercised control condition. EX-DEF, morning after prolonged exercise without carbohydrate replacement and thus during carbohydrate deficit; EX-BAL, morning after prolonged exercise with sufficient carbohydrate replacement to restore carbohydrate and energy balance following exercise. Large, medium and small VLDL are defined as 55 – 260 nm, 43 – 55 nm and 29 – 43 nm diameters respectively with data also presented for 70 – 120 nm range (subset of large). IDL, large LDL and small LDL are defined as 23 – 27 nm, 21.2 – 23 nm and 18 – 21.2 nm diameters, respectively. Large HDL, medium HDL and small HDL are defined as 8.8 - 13 nm, 8.2 – 8.8 nm and 7.3 – 8.2 nm diameters respectively. Differences assessed using one-way repeated measures ANOVA followed by LSD post-hoc. *p < 0.05 vs CON; **p < 0.05 compared to EX-DEF; □ data log-transformed prior to statistical analysis.

The 24-part VLDL subfraction analysis (Figure 1) indicates a number of VLDL triglyceride mass peaks, with the greatest mass of TG in particles of ~ 70–120 nm in diameter. In this 70–120 nm size range, a subset of large VLDL, the TG mass was lower (p < 0.05) in EX-DEF but not in EX-BAL compared to CON (Figure 1, Table 1). The subfraction analysis also reveals that in the medium sized particles, the TG mass was lower (p < 0.05) both in EX-DEF and EX-BAL compared to CON (Figure 1, Table 1). A small TG mass peak of ~225 nm was also obvious in all three trials (Figure 1).

### Exercise, LPL activity and lipoprotein particles

**Figure 2:**
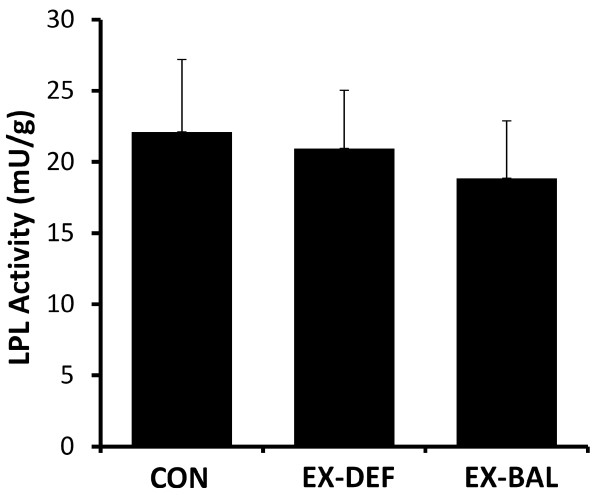
Skeletal muscle lipoprotein lipase activity in CON, EX-DEF and EX-BAL.

LPL activity was not different in CON vs EX-DEF (n = 7, p = 0.74) or CON vs EX-BAL (n = 7, p = 0.50) (Figure 2). The *intra*-individual responses to exercise from CON were highly correlated in EX-DEF and EX-BAL (rho = 0.93, p < 0.05). There were inverse correlations that were either significant or approaching significance between the % change in LPL activity in CON vs. EX-DEF and the change in serum TG (rho = −0.89, p < 0.05), total VLDL-TG (rho = −0.79, p < 0.05), large VLDL-TG (rho = −0.75, p = 0.052), large VLDL particle concentration (rho = −0.71, p = 0.07), mean VLDL particle size (rho = −0.89, p < 0.05), small HDL particle concentration (rho = −0.75, p = 0.052) and small LDL particle concentration (rho = −0.93, p < 0.05). In CON, LPL activity was inversely correlated with serum TG, total VLDL-TG, total, large and small VLDL particle concentration and mean VLDL particle size (Table 2). There were few associations between LPL activity and lipoprotein particles in EX-DEF and EX-BAL (Table 2).

**Table 2:** Association of skeletal muscle lipoprotein lipase activity with lipid and lipoprotein particles in CON, EX-DEF and EX-BAL

**Lipid /Lipoprotein parameters**	**CON**	**EX-DEF**	**EX-BAL**
Serum TG (mg/dL) (biochemical)	−0.98*	`-0.36	−0.56
VLDL-TG (mg/dL)	−0.81*	−0.21	−0.68
Total VLDL particles (nmol/L)	−0.76*	+0.46	+0.25
Large VLDL particles (nmol/L)	−0.95*	−0.61	−0.68
Medium VLDL particles (nmol/L)	−0.24	−0.36	−0.25
Small VLDL particles (nmol/L)	−0.86*	+0.93*	+0.29
IDL particles (nmol/L)	−0.57	−0.14	−0.21
LDL particles (nmol/L)	−0.41	+0.14	−0.18
Small LDL particles (nmol/L)	−0.10	−0.36	−0.39
HDL particles (μmol/L)	+0.25	−0.04	−0.11
Large HDL particles (nmol/L)	+0.72*	+0.25	+0.23
Mean VLDL size (nm)	−0.90*	−0.79*	−0.61
Mean HDL size (nm)	+0.90*	+0.38	+0.05
Mean LDL size (nm)	+0.30	+0.11	−0.16

*p < 0.05; for definitions see Table 1.

## Discussion

By using new NMRS analytical techniques to better resolve the concentration of lipoproteins of different sizes, this study has undertaken one of the most detailed characterizations to date of the effects of a single session of exercise on VLDL lipoprotein particle concentration and TG content. This involved two exercise trials, one with and one without carbohydrate refeeding. This allowed us to distinguish the effects of prior exercise on lipoproteins when there was an exercise-induced carbohydrate deficit and glycogen depletion (EX-DEF) from a very different metabolic situation when the post-exercise testing was associated with carbohydrate and energy balance (EX-BAL). In general, the findings reveal a high level of differential regulation between different sizes of VLDL with respect to exercise and feeding.

The NMRS technique provides new insights about the concentration of lipoprotein particles with more clarity than possible from biochemical methods. Although there are far more apoB-100 containing VLDL particles in the smaller sized range of the continuum (~29 – 43 nm), the TG content of VLDL per particle is considerably less than for larger particles. Particles in the 70–120 nm range (subset of large range) contain ~ 44% of the TG carried by VLDL particles. The present findings clearly indicate that the intensity and type of exercise utilized in this study which was glycogen depleting, was specifically impacting VLDL in a relatively narrow moderate-large size range (~43–120 nm). The smaller particles were resistant to reduction by either exercise condition, despite both the high intensity exercise (70-100% maximal aerobic power) and profound skeletal muscle glycogen depletion. Interestingly, this same type of lack of response was also seen in the very large particles. Within the “exercise responsive” range (~43–120 nm), all fractions did not respond identically. An important new insight was that the reduction in the TG mass in the 70 – 120 nm subset of large VLDL, the size range with the greatest TG mass, was primarily due to an exercise-induced carbohydrate deficit. This novel finding now explains why there was not a decrease in serum TG in EX-BAL (as measured with classical biochemical methods) on the morning following a very prolonged and intense bout of exercise. In contrast, the VLDL-TG in the medium range was unresponsive to the post-exercise carbohydrate feeding and was thus reduced in both exercise conditions, demonstrating an effect of exercise *per se*. A TG mass peak of ~ 225 nm is obvious in all three trials though it is possible that this represents the late entry of intestinally-derived chylomicrons into the circulation. These varied VLDL responses to different exercise conditions are not easy to explain mechanistically by any single factor such as increased skeletal muscle lipoprotein lipase activity.

Few previous studies have examined the influence of acute exercise on VLDL subfractions. In general agreement, Magkos et al. [14] reported a 36% (non significant) and 53% reduction in their NMRS analysis of relatively large and medium-sized VLDL particles respectively, with no change in smaller VLDL particle concentration. In characterizing the influence of acute exercise on VLDL subclasses using ultracentrifugation, Gill et al. [23] found that there was a greater absolute and percentage decrease in TG in the “large” VLDL_1_ subclass (Sf 60–400) but there was also a reduction in the “small” VLDL_2_ subclass (Sf 20–60). Although papers reporting particle size in various Svedberg flotation density ranges are sparse in the literature, data from one helpful paper [24] suggests the VLDL_1_ subclass (Sf 60–400) reported in the literature encompasses a number of particle size ranges above ~35 nm, essentially our large and medium particles, with the VLDL_2_ subclass (Sf 20–60) encompassing particles in the middle and lower end of our small range. The results of the present study are therefore in agreement with the findings of Gill et al. [23] with respect to VLDL_1_, though confirming an exercise effect in a number of subfractions within this range, but are not in agreement with their findings with respect to VLDL_2_. Gill et al. [25] have also suggested previously that there may be independent metabolic regulation of the VLDL_1_ and VLDL_2_ subclasses. The results of the present study suggest that there may be independent regulation of different VLDL pools even within the VLDL_1_ range.

Large VLDL may be more important for risk than medium and small VLDL [9]. In theory, the more efficient metabolism of large VLDL as occurred in EX-DEF should lead to anti-atherogenic changes in four important lipoprotein parameters, an increase in large HDL concentration with increased mean HDL size and a decrease in small LDL concentration with increased mean LDL size [9]. However, there were no changes in HDL, LDL or IDL subfractions or mean particle size in either exercise condition in the present study. Perhaps this is not surprising given the considerably longer half-life of LDL and HDL particles in the circulation [26]. Changes in VLDL metabolism over a number of days may be necessary to influence LDL and HDL. Magkos et al. [14] reported an acute exercise increase in pro-atherogenic IDL in conjunction with a more efficient breakdown of VLDL particles. In contrast, IDL were directionally though not significantly lower in the present study.

Previously published postprandial data from this cohort [5] and from two other groups [4,27] show the hypotriglyceridemic effects of exercise to be attenuated or abolished with post-exercise feeding to restore energy and CHO balance. The results of one of these studies [27] suggest that post-exercise hypotriglyceridemia may relate more to a CHO deficit than an energy deficit. However the extent to which individuals replenish energy or CHO immediately post-exercise in “real world” settings is open to question. Using a different research design, prior exercise reduced triglycerides during a postprandial period that allowed *ad libitum* feeding, even though energy intake was increased as a result of the *ad libitum* feeding regime [28]. In the present study, high glycaemic (GI) index CHO was fed post-exercise in EX-BAL, intended to restore muscle glycogen to CON values without supercompensation The hypertriglyceridemic effects of high GI diets are well known [29]. This high GI refeeding in EX-BAL exerted its effects on large VLDL particle concentration only with no change in medium or small VLDL or any other lipoprotein particles compared to EX-DEF. Particle concentration changes in this TG-rich fraction have the greatest potential to influence total serum TG. It is unclear if low GI CHO or high fat feeding to maintain a CHO deficit while restoring energy balance, would have influenced the lipoprotein particle distribution similarly. It should be noted that medium VLDL-TG was lowered following acute exercise by nearly 50% even in the presence of high GI CHO feeding. However, there was insufficient statistical power to detect a difference in serum TG between CON and EX-BAL, as medium sized VLDL particles accounted for only ~12% of total circulating TG.

Skeletal muscle LPL activity was not different from CON in either exercise condition, despite the vigorous, exhausting and glycogen depleting nature of the exercise. This study provides evidence that LPL activity is not necessarily a response required to explain the influence of either exercise or post-exercise CHO refeeding on serum lipids and lipoproteins. Indeed a review of the available literature does not provide compelling evidence that acute exercise consistently increases LPL activity. Studies have found LPL activity to be increased [30-32] but also unchanged [33,34] in the early post-exercise period, increased [12] and unchanged [11] on the morning after exercise. Other studies have reported increases in LPL activity that are restricted to certain muscle fibre types [19], timepoints [35], genders [36] and apo E haplotypes [37]. In this recent genetic study [37], exercise training actually reduced post-heparin LPL activity in the most common apo E haplotype, though this did not prevent a reduction in serum TG.

The results of the present study do not rule out a contributing influence of LPL on lipid and lipoprotein particles, especially under other conditions or types of subjects. Although mean LPL activity was unchanged, the changes in serum TG, VLDL-TG, VLDL size and large VLDL particle concentration between CON and EX-DEF were inversely correlated with the change in LPL activity, a phenomenon also observed in other similarly designed studies [11,38]. It appears that this association was driven by changes in large VLDL particles. A recent review [10] has described in more detail than possible here why LPL activity may possibly contribute, but not be a primary factor for TG lowering after exercise. Current research attention has in part begun focusing on the issues related to VLDL compositional changes to account for the effects of acute exercise on VLDL metabolism [13], with the secretion of fewer but TG-richer VLDL_1_ particles by the liver, potentially increasing interaction with LPL in the periphery. In the present study, large and medium VLDL particle concentrations were indeed lower in EX-DEF with small VLDL and IDL particle concentrations unchanged, though these reductions do not confirm such a secretional change. Of interest, the correlations suggest that exercise-induced increases in LPL activity may also have the potential to influence HDL and LDL metabolism and in particular to decrease the concentration of pro-atherogenic small LDL particles. The results of this study also highlight a role for skeletal muscle LPL activity in regulating serum TG and VLDL and HDL lipoprotein particles in the non-exercised control state. In the exercised state, few associations were evident, thus the importance of LPL appears to be overridden by other unknown factors.

In summary, this study has in much greater detail than hitherto characterized the influence of prolonged acute exercise on the VLDL particle profile. On the morning after prolonged exercise without postexericse CHO feeding, large VLDL are considerably reduced, in terms of particle concentration and the corresponding TG mass. However, this exercise effect on large VLDL, is only apparent when a negative CHO balance is maintained into the post-exercise period. In contrast, medium VLDL do not increase with CHO intake post-exercise. These effects are only obvious with this NMRS technique.

## Competing interests

The authors declare that they have no competing interests.

## Authors’ contributions

MH, NMM and DJO’G were responsible for the research design. MH, TWZ, NMcC and BPC were involved in data collection and the biochemical analyses. MH, MTH, TWZ and NMM were involved in data analysis and manuscript writing. TWZ and MTH conceptualized the focus on the lipoprotein particle distribution, were responsible for the analytical NMRS strategy, and performed the skeletal muscle LPL assays. All authors approved the final submission.
